# Autistic Traits and Brain Activation during Face-to-Face Conversations in Typically Developed Adults

**DOI:** 10.1371/journal.pone.0020021

**Published:** 2011-05-27

**Authors:** Masashi Suda, Yuichi Takei, Yoshiyuki Aoyama, Kosuke Narita, Noriko Sakurai, Masato Fukuda, Masahiko Mikuni

**Affiliations:** Department of Psychiatry and Neuroscience, Gunma University Graduate School of Medicine, Gunma, Japan; The University of Hong Kong, Hong Kong

## Abstract

**Background:**

Autism spectrum disorders (ASD) are characterized by impaired social interaction and communication, restricted interests, and repetitive behaviours. The severity of these characteristics is posited to lie on a continuum that extends into the general population. Brain substrates underlying ASD have been investigated through functional neuroimaging studies using functional magnetic resonance imaging (fMRI). However, fMRI has methodological constraints for studying brain mechanisms during social interactions (for example, noise, lying on a gantry during the procedure, etc.). In this study, we investigated whether variations in autism spectrum traits are associated with changes in patterns of brain activation in typically developed adults. We used near-infrared spectroscopy (NIRS), a recently developed functional neuroimaging technique that uses near-infrared light, to monitor brain activation in a natural setting that is suitable for studying brain functions during social interactions.

**Methodology:**

We monitored regional cerebral blood volume changes using a 52-channel NIRS apparatus over the prefrontal cortex (PFC) and superior temporal sulcus (STS), 2 areas implicated in social cognition and the pathology of ASD, in 28 typically developed participants (14 male and 14 female) during face-to-face conversations. This task was designed to resemble a realistic social situation. We examined the correlations of these changes with autistic traits assessed using the Autism-Spectrum Quotient (AQ).

**Principal Findings:**

Both the PFC and STS were significantly activated during face-to-face conversations. AQ scores were negatively correlated with regional cerebral blood volume increases in the left STS during face-to-face conversations, especially in males.

**Conclusions:**

Our results demonstrate successful monitoring of brain function during realistic social interactions by NIRS as well as lesser brain activation in the left STS during face-to-face conversations in typically developed participants with higher levels of autistic traits.

## Introduction

Autism is a developmental disorder characterized by impaired social interactions and communication in addition to restricted and repetitive behaviour. In recent years, the concept of autism spectrum disorders (ASD) has been proposed; it hypothesizes a wide range of symptoms resembling autism, such as those demonstrated in Asperger syndrome and pervasive developmental disorder not otherwise specified. According to this concept, disorders across the spectrum are believed to have common biological bases. Postmortem and structural magnetic resonance imaging (MRI) studies have highlighted the prefrontal cortex (PFC), including the medial PFC, the superior temporal sulcus (STS), the amygdala, the anterior cingulate cortex, the fusiform gyrus, the thalamus, and the cerebellum as pathological substrates for ASD [1].

Many functional neuroimaging studies of ASD focusing on impaired social interactions and communication have been conducted using functional MRI (fMRI). By employing task stimuli related to social cognitive modules, such as face recognition, visual motion processing, the theory of mind, and eye-gaze perception, these studies have implicated several brain regions in the pathogenesis of autism, including the STS and the fusiform gyrus for face processing [2], [3]; the PFC, including the medial prefrontal cortex, for mentalising and person perception [4], [5]; the temporoparietal junction [6]; and the amygdala for threat detection, emotion recognition, and complex social judgments [7], [8]. Moreover, these studies have provided a foundation for understanding neural mechanisms underlying social deficits in ASD. However, fMRI has methodological constraints for studying brain mechanisms underlying social cognition. For example, participants are required to lie on a bed in a small, noisy gantry during examination, a condition that is upsetting to many people, including those with autism. Due to this limitation, most previous studies have necessarily been conducted in an unusual and unrealistic way, such as using pictures or computer graphics images shown on a computer monitor as task stimuli. A functional brain imaging methodology that enables monitoring of brain activation in a more natural setting might well offer more informative data from more realistic social interactive situations, such as having an interview with another person, which is impossible with fMRI because of its methodological constraints.

Near-infrared spectroscopy (NIRS) is a recently developed functional brain imaging technique that involves emission of near-infrared light that can be detected through the scalp [9]. NIRS allows monitoring of cerebral blood volume (CBV) changes in the neocortex as indicated by increased oxygenated haemoglobin concentrations ([oxy-Hb]) and decreased deoxygenated haemoglobin concentration ([deoxy-Hb]) using a small apparatus, although certain measurement concerns remain, such as the effect of blood flow in the scalp or the difficulty in determining the exact length of the light path for each subject. NIRS has some methodological limitations as well, such as a low spatial resolution (approximately 3 cm, which is nearly equal to 1 gyrus of the brain) and an inability to assess deep brain structures. Nevertheless, when an NIRS probe is placed on the head in one of the 10–20 standard electroencephalography electrode positions, the cerebrocranial correlation is considered to vary within 1 cm; therefore, correspondence at the level of the gyrus is not affected [10].

Despite these methodological limitations, NIRS enables brain activity measurement in a more natural setting compared with other functional brain imaging techniques. Subjects can undergo NIRS examination in a seated position, with their eyes open, while speaking, and without any noise or pain. These characteristics of NIRS are considered to be particularly suitable for social interaction studies. Thus far, NIRS has successfully been demonstrated for monitoring brain function in healthy participants during delicate and/or subjective experiences, such as subjective sleepiness and psychological fatigue [11], [12] and in patients with psychiatric disorders who are sensitive to the experimental environment [13]–[16]. In short, NIRS has certain distinct advantages, such as complete non-invasiveness, lack of restriction of body movement, and the small size of the apparatus, but it is not able to detect signals within the deep brain structure and has a low spatial resolution of approximately 1 gyrus.

In this study, we used NIRS to monitor brain activation in healthy seated participants during conversations to examine social cognition in a natural setting. Such an approach may further our understanding of brain activity during social interactions in everyday life and of associations between multiple social cognitive modules in realistic situations. We further investigated the relationship between brain activation in the PFC and STS regions during face-to-face conversations and, because the severity of characteristics of ASD is posited to lie on a continuum that extends into the general population, we evaluated autistic traits in typically developed adults [17], [18]. To determine the extent to which adults of average intelligence display characteristics associated with ASD, Baron-Cohen et al. developed a self-administered questionnaire, the Autism-Spectrum Quotient (AQ) [19]. We hypothesized that face-to-face conversations would activate the PFC as well as the STS (since both areas are involved in social cognition) and that variations in autistic traits in the typically developed participants would be correlated with brain activation during face-to-face conversations.

## Methods

### Participants

Twenty-eight healthy volunteers participated in this study (14 males and 14 females; average age, 26.4 years; standard deviation [SD], 3.0; range, 23–35). The participants in this study were medical interns at Gunma University Hospital and students at Gunma University Faculty of Medicine. All were right-handed and had no history of any major psychiatric disorder, autism, neurological disorder, substance abuse, head injury, or major physical illness. Moreover, they were not on any psychotropic medications at the time of the study. These participants had also been included in our previous study [16], and their autistic traits were assessed using the Japanese version of the AQ [19], [20]. This study was approved by the Institutional Review Board of the Gunma University Graduate School of Medicine. Written informed consent was obtained from all participants prior to the study.

### Activation Task

**Figure 1:**
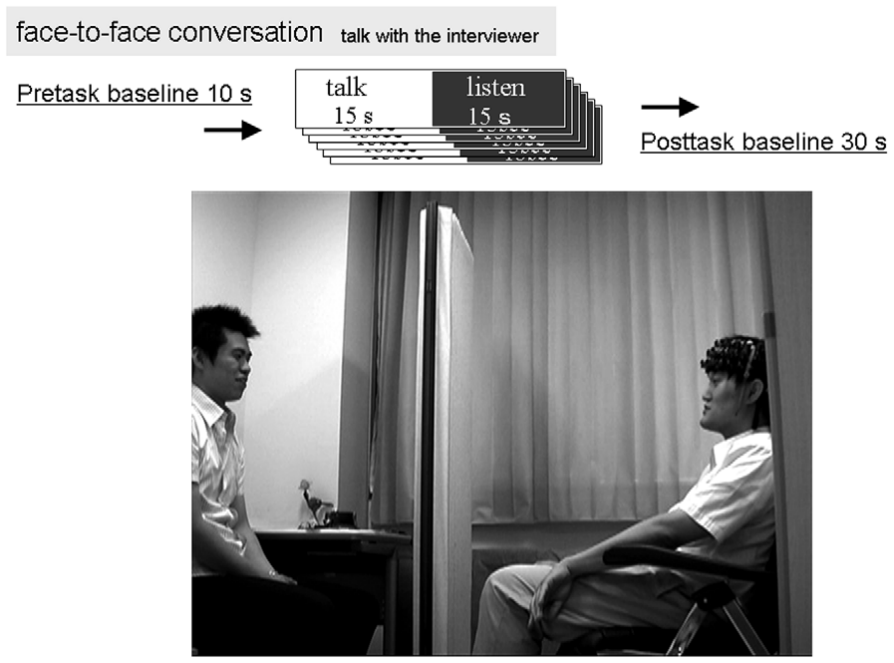
Task procedures and setting. The participants were required to talk with the interviewer seated in front of them during the task segment, in which they faced each other and sat on chairs. The task period consisted of 6 cycles of a 30-s conversation, with total conversations as long as 180 s. Before beginning and after finishing the task, the participant and the interviewer were separated by a partition so that they could not see each other; during the experiment itself, the partition was removed. As shown in the photograph, the participant wearing the near-infrared spectroscopy probe on his forehead sat on the right, whereas the interviewer sat on the left.

We employed 2 types of activation tasks: a conversation condition and a control condition. The order of the 2 tasks was counterbalanced among the participants. The participants sat on comfortable chairs in a room throughout the measurement process (Figure 1). Direct sunlight was shut out by a curtain.

#### Conversation condition

The task was designed to simulate ordinary conversations in everyday life, albeit in an experimental setting. Each participant and an interviewer sat face to face 1 m apart on comfortable chairs in a sunlit room with their eyes open, and the NIRS probes were placed on the participant's frontal and temporal regions. Before beginning and after finishing the experiment, the participant and the interviewer were separated by a partition so that they could not see each other. The partition was removed during the experiment.

The experiment consisted of 3 periods: pre-task, task, and post-task. During the task period, the participant was required to talk with the interviewer in front of them. To avoid qualitative and quantitative differences among the conversations, the participants were instructed to make conversation during the task period in accordance with the following 2 criteria. First, the time course of the conversation was settled a priori: the subject and the interviewer were to speak in turn, in that order, every 15 s; this was accomplished via verbal cues from the experimenter every 5 s. The task period consisted of 6 30-s conversation cycles, with the entire conversation lasting as long as 180 s. Second, the theme of the conversation was limited to anything related to food. The theme of the conversation was limited to food because in initial experiments to test the conversational task, this was one of the most popular topics among all of the participants and was relatively easy to discuss with a person upon meeting for the first time. The interviewers were 3 male psychiatrists who were not acquainted with the participants. During the pre-task and post-task periods, the participants were instructed to repeat the syllables /a/, /i/, /u/, /e/, and /o/ (the Japanese counterparts of English vowel sounds) to exclude the effect of phonation and stabilize the baseline conditions. Using videotape, the images and voices of the subjects and interviewers were recorded during the experiment for further analysis.

Task performance during the conversation was evaluated in 3 ways. First, the amount of discussion by the participants was evaluated quantitatively as speaking time, which corresponded to the length of the participants' speech measured using the recorded videotape. Second, the content of the conversation was evaluated qualitatively in terms of receiving and sending aspects. The receiving aspect (RS) indicated the appropriateness of the response within the context of the conversation: the subject's replies to the preceding statements by the interviewer were scored as (1) appropriate, (2) partially appropriate, (3) partially inappropriate, and (4) inappropriate. The sending aspect (SS) indicated the productivity of new topics: the subject's questions to the interviewer were scored as (1) a completely new topic, (2) a partially new topic, (3) nearly the same topic, and (4) not a new topic. Third, the expressiveness of the subjects was evaluated by observation according to the Broader Phenotype Autism Symptoms Scale [21]. Expressiveness consists of 4 dimensions: eye gaze (an individual's eye contact both when listening and speaking or when otherwise interacting with the examiner), social smiling (an individual's response to the examiner's smiles), facial expressions (the range and appropriateness of facial expressions), and prosody (whether an individual exhibits atypical rate, rhythm, volume, and/or intonation of speech). Each dimension was scored as (1) normal, average, or typical functioning for a person of that age and life circumstance; (2) on the lower end of the average range or somewhat lower than most people, but not significantly impaired; (3) outside the normal range, definitely below average, or impaired; or (4) far outside the normal range, well below average, or significantly impaired.

#### Control condition

To examine brain activation and artefact contamination induced by phonation only, a control task was conducted in addition to the experimental task. The subjects were instructed to repeat meaningless syllables such as ‘a’, ‘ka’, ‘sa’, ‘ta’, and ‘na’ during their turns to speak in the task period. All subjects were able to repeat such syllables without interruption.

### Near-infrared Light Spectroscopy Measurement

In this study, changes in [oxy-Hb] and [deoxy-Hb] were measured using a 52-channel (Ch) NIRS machine (Hitachi ETG-4000). Absorption at 2 wavelengths of near-infrared light (780 and 830 nm) was measured, from which [oxy-Hb] and [deoxy-Hb] were calculated, respectively. As an index of CBV changes, [oxy-Hb] changes were evaluated. The distance between the pairs of emission and detector probes was 3.0 cm, and the machine was considered to measure depths of 2–3 cm below the scalp; that is, at the surface of the cerebral cortex [22], [23].

The probes of the NIRS machine were placed on the participant's frontal region (Figure S1 online). The frontal probes measured [Hb] changes at 52 measurement points over a 6×30-cm area. We used a 3×11 probe holder for the Hitachi ETG-4000 with 17 light sources and 16 light detectors, with the lowest probes positioned along the Fp1–Fp2 line, in accordance with the international 10/20 system used in electroencephalography.

Absorption of near-infrared light was measured at a time resolution of 0.1 s. The obtained data were analyzed using the ‘integral mode’. The pre-task baseline was determined as the mean of the last 10 s of the 30-s pre-task period, the post-task baseline was determined as the mean of the last 10 s of the 30-s post-task period, and linear fitting was applied to the data between these 2 baselines. The moving average method was used to exclude short-term motion artefacts in the analyzed data (moving average window: 5 s).

### Data Analyses

The dependence of autistic traits on the participants' age and gender was examined using multiple regression analyses employing the AQ score as a dependent variable and age and gender as independent variables. The relationship between autistic traits and task performance during face-to-face conversations was also examined using multiple regression analyses employing 3 indices of task performance as dependent variables: speaking time, the score for qualitative evaluation of the RSs, and the score for qualitative evaluation of the SSs. AQ score, age, and gender were the independent variables.

The waveforms of [Hb] changes for all 52 Chs under conversation and control conditions were calculated for all participants. NIRS data from Chs that clearly contained artefacts, as determined by close observation of the subjects (Chs 1–21), were excluded from further analyses. The most common cause of these artefacts was NIRS probe drift due to the presence of hair. Probes placed on an area with a lot of hair are difficult to adequately fasten onto the head and can be easily displaced.

First, the average [Hb] changes during the 2 tasks in the 4 regions of interest were calculated: right PFC (Chs 25, 26, 35, 36, and 47), right STS (Chs 22, 32, 33, 43, and 44), left PFC (Chs 27, 28, 38, 39, and 48), and left STS (Chs 31, 41, 42, 51, and 52). These were identified in accordance with correspondences between the NIRS Chs and measurement points on the cerebral cortex as determined by the virtual registration method in which structural information from an anatomical database is used to obtain estimates of Ch positions in a standardized stereotaxic 3D brain atlas (Figure S1 online) [24]. There were 3 light sources and 3 detectors in the PFC, and 3 light sources and 2 detectors in the STS. Averaged [oxy-Hb] changes during the 180-s task segment across 5 Chs in each of 4 regions were analyzed by four-way repeated-measures analysis of covariance (ANCOVA) with task (conversation or control) and gender as the inter-individual independent variables, laterality (right or left) and region (PFC or STS) as the intra-individual independent variables, and age as the covariate. This was followed by post hoc *t*-tests. We analyzed age as the covariate because [oxy-Hb] changes over the frontal lobe during a cognitive task were significantly correlated with age in our previous NIRS study [14].

We then investigated the relationship between [Hb] changes during face-to-face conversations and autistic traits of the participants. A simple correlation of [Hb] changes under conversation and control conditions in the 4 regions of interest with AQ score was conducted. In addition, the relationships between [Hb] changes and autistic traits by gender were analyzed to test a theory described by Baron-Cohen as ‘extreme male brain’, which attempts to account for differences in autistic traits between the 2 genders [25].

Finally, we investigated relationships between activation in the 4 regions of interest. Simple correlations were examined between regional CBV changes among the 4 regions of interest, right PFC, right STS, left PFC, and left STS, among all the participants and by gender.

## Results

### Characteristics of Participants and Behavioural Data

The AQ scores and task performances of the participants are shown in Table S1 (online). An AQ cut-off score of ≥32 is often used to identify a person as having autism; in the present study, 1 subject scored 33 (subject no. 14, Table S1 online). Task performances during the face-to-face conversations were not correlated with AQ scores, age, or gender, and the AQ scores were not significantly correlated with age or gender.

### [Hb] Changes during Face-to-Face Conversations

**Figure 2:**
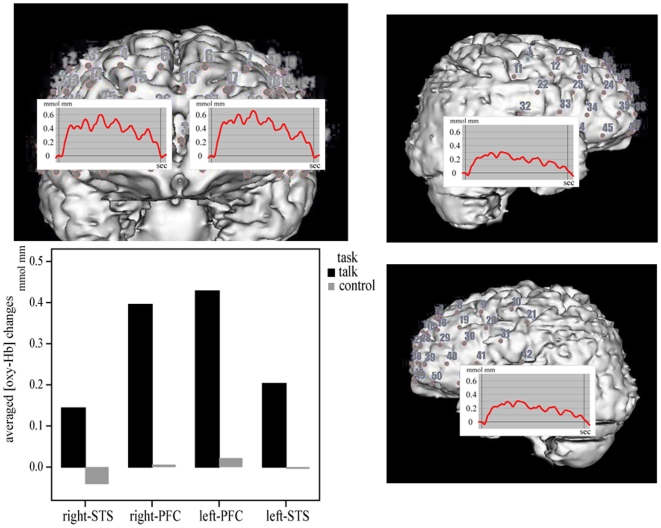
Grand-averaged waveforms of oxygenated haemoglobin concentrations ([oxy-Hb]) under task and control conditions. Grand-averaged waveforms of [oxy-Hb] under conversation conditions (red line) and during the entire 180-s task segment (between each pair of vertical lines) under conversation conditions (black bar) and control conditions (gray bar) as measured by near-infrared spectroscopy in 4 regions of interest: right prefrontal cortex (PFC), right superior temporal sulcus (STS), left PFC, and left STS.

Average [oxy-Hb] changes during the 180-s task segment and grand-averaged waveforms across 5 Chs in 4 regions are shown in Figure 2. The average [oxy-Hb] changes during the 180-s task segment in 4 regions are shown in Table 1. Four-way repeated-measures ANCOVA showed that the main effects of the task (*F*[1,51] = 59.7, *P*<0.001) and region (*F* = 6.2, *P* = 0.016) were significant, but the main effects of laterality (*F*[1,51] = 0.9, *P* = 0.33) and gender (*F*[1,51] = 0.7, *P* = 0.42) were not. The significant main effects of task and region indicate significantly greater [oxy-Hb] increases under the conversation condition than under the control condition, and significantly greater [oxy-Hb] increases in the PFC than in the STS. Four-way repeated-measures ANCOVA also showed significant two-way interactions of task by region (*F*[1,51] = 13.3, *P* = 0.001) and task by gender (*F*[1,51] = 5.1, *P* = 0.028); the other interactions were not significant. These significant two-way interactions indicate that [oxy-Hb] increased predominantly in the PFC under the conversation condition but not under the control condition. In addition, there were greater [oxy-Hb] increases in males than in females under the conversation condition, but the reverse was observed under the control condition.

**Table 1:** Averaged [oxy-Hb] and [deoxy-Hb] changes during conversation condition and control condition.

			right STS		right PFC		left PFC		left STS
Conver.	[oxy-Hb]		0.14 (0.22)	0.40 (0.33)	0.43 (0.31)	0.20 (0.10)			
	[deoxy-Hb]	−0.05 (0.14)	−0.05 (0.14)	−0.04 (0.07)	−0.04 (0.13)				
Control	[oxy-Hb]		−0.4 (0.11)	0.01 (0.08)	0.02 (0.08)	−0.003 (0.08)			
	[deoxy-Hb]	−0.002 (0.03)	−0.003 (0.07)	0.007 (0.03)	−0.02 (0.07)				
								mean (SD)	

Conver., conversation condition; Control, control condition; [oxy-Hb], oxygenated hemoglobin; [deoxy-Hb], deoxygenated hemoglobin; STS, superior temporal sulcus; PFC, prefrontal cortex.

The average [deoxy-Hb] changes during the 180-s task segment in 4 regions are shown in Table 1. Four-way repeated-measures ANCOVA showed a significant main effect of the task (*F*[1,51] = 6.3, *P* = 0.016), but did not show any other significant main effects or interactions. The main effect of task indicates significantly greater [deoxy-Hb] decreases under the conversation condition than under the control condition.

### Relationships between Brain Activation and AQ Scores

**Figure 3:**
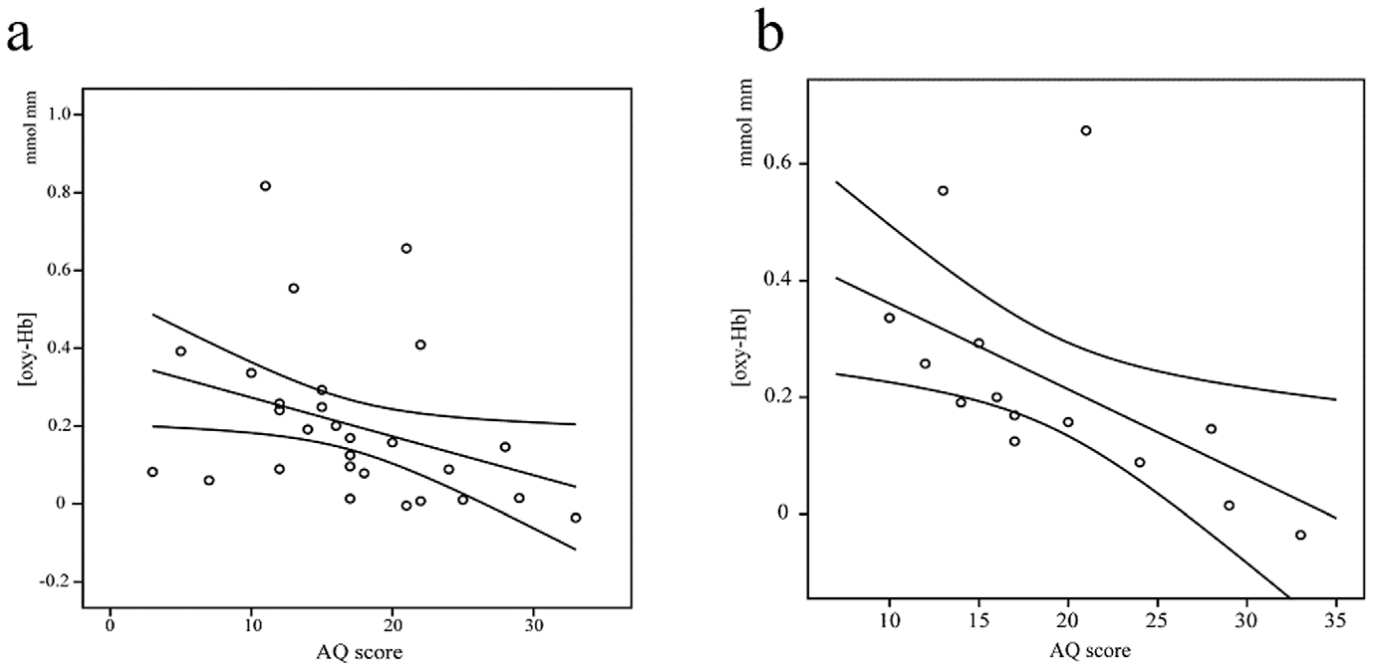
Correlation between autistic traits assessed by the Autism-Spectrum Quotient (AQ) and brain activation. **a:** Total AQ score vs. the oxygenated haemoglobin concentration ([oxy-Hb]) as measured by near-infrared spectroscopy (NIRS) in the left superior temporal sulcus (STS) among all participants. **b:** Total AQ score in male subjects vs. [oxy-Hb] as measured by NIRS in the left STS.

Correlations between AQ scores and [oxy-Hb] changes under the conversation condition in the 4 regions of interest are shown in Table 2. AQ scores were correlated with [oxy-Hb] changes in the left STS (rho = −0.460, *P* = 0.01; Figure 3a) but not with the other regions under the conversation condition. There were no significant correlations between [oxy-Hb] changes and AQ scores under the control condition.

**Table 2:** Correlation between AQ score and [oxy-Hb].

AQ vs.	right STS	right PFC	left PFC	left STS
rho	−0.063	−0.17	−0.136	−.460*
P value	0.75	0.388	0.489	.014*

AQ, Autism Spectrum Quotient; oxy-Hb, oxygenated hemoglobin; STS, superior temporal sulcus; PFC, prefrontal cortex.

Although the communication subscore correlated with [oxy-Hb] changes in the left STS under the conversation condition (rho = −0.429, *P* = 0.02), the correlation was no longer significant after Bonferroni correction for multiple correlations. The corrected significance level was set at *P*<0.0125 based on the following rationale: we excluded the 16 [deoxy-Hb] comparisons from the requisite Bonferroni divisor; physiologically, it is understood that the cerebrovascular response exerts a much lesser effect on [deoxy-Hb] than on [oxy-Hb]. The 4 remaining control-task comparisons were excluded on the basis of prior expectation of an effect only in the conversation task.

AQ scores were significantly correlated with [oxy-Hb] changes in the left STS in male participants (rho = −0.730, *P* = 0.003; Figure 3b) but not in female participants (rho = −0.434, *P* = 0.12) under the conversation condition (after Bonferroni correction for multiple correlations, *P*<0.006). However, there was no significant difference between the 2 coefficients of correlation (*Χ* ^2^ = 1.8161, df = 1, *p* = 0.1778).

### Associations between Activation in the 4 Regions of Interest

For [ox-Hb] changes during face-to-face conversation, there was only 1 significant correlation between the right PFC and left PFC (*r* = 0.927, *p*<0.001); the other correlations were not significant after Bonferroni correction for multiple correlations (*P*<0.008). In males, there was only 1 significant correlation between the right PFC and left PFC (*r* = 0.941, *p*<0.001), similar to the mixed group. However, in females, there were additional significant correlations between the left STS and right PFC (*r* = 0.716, *p* = 0.004) and the left STS and left PFC (*r* = 0.682, *p* = 0.007), in addition to the right PFC and left PFC (*r* = 0.890, *p*<0.001). These results indicate that there were strong positive correlations between bilateral PFC activations during conversation; however, in females, there were additional positive correlation between left STS activation and PFC activation. For [oxy-Hb] changes under control conditions, there was only 1 significant correlation between the right PFC and left PFC (mixed group: *r* = 0.862, *p*<0.001; males: *r* = 0.864, *p*<0.001; females: *r* = 0.854, *p*<0.001); the other correlations were not significant.

For [deoxy-Hb] changes, there was only 1 significant correlation between the right PFC and left PFC (mixed group: *r* = 0.658, *p*<0.001; males: *r* = 0.721, *p* = 0.004; females: not significant) during conversation; the other correlations were not significant.

## Discussion

In this study, we examined brain activation during face-to-face conversations by measuring regional CBV (rCBV) increases using NIRS. The results demonstrated that 1) face-to-face conversation was accompanied by significant bilateral rCBV increases in the PFC and STS regions, with greater increases in the PFC than in the STS, 2) there were strong positive correlations between bilateral rCBV increases in the PFC in both genders, but there were additional positive correlations between left STS activation and bilateral PFC activations in females, and 3) AQ scores of typically developed (non-ASD) participants were negatively correlated with rCBV increases in the left STS region during face-to-face conversation, especially in male participants.

### Conversation Performances and Autistic Traits

Task performance, i.e. the amount of speech and expression of the participants, was not correlated with autistic traits. This unexpected absence of correlation may be because only 1 subject was above the AQ cut-off. Alternatively, since all of the participants in this study were healthy and socially well-functioning subjects and were able to perform the conversation task quite easily (Table S1 online), there may have been subject selection bias. If subjects with higher AQ scores or lower levels of social functioning had been included, we might have seen significant correlations between task performance and autistic traits.

### Brain Activation during Face-to-Face Conversation

Brain activation in the PFC and STS are assumed to represent the output and input aspects of conversation, respectively [26], [27]. In our study, the former was greater than the latter only under face-to-face conversation conditions. Because the NIRS Chs in this study detected dorsolateral but not medial PFC activities, the obtained PFC activations are assumed to reflect executive function mediated by the dorsolateral PFC [28]. The nature of the task employed in this study may explain the greater activation in the PFC (reflecting executive functions such as planning, problem solving, and working memory) than in the STS (reflecting social cognition): the subjects were required to actively generate conversation by broaching topics and asking questions. Therefore, the greater activation in the PFC than in the STS in this study is possibly related to the task characteristics.

Based on the results of correlations among activation in the regions of interest, we speculate that the male participants may have conducted face-to-face conversation using mainly the bilateral PFC, corresponding to executive functions such as planning and problem solving. However, the female participants may have conducted face-to-face conversation using both the STS, corresponding to social cognitive function, and the PFC in a coordinated manner. These results are consistent with empirical knowledge relating to differences in conversation styles by gender; females tend to be more ‘empathising’ and males tend to be more ‘systemising’ [29].

### Left STS and Autistic Traits

AQ scores were negatively correlated with rCBV increases during face-to-face conversations only in the left STS region; the typically developed (non-ASD) subjects with higher levels of autistic traits demonstrated lesser brain activation, especially in males. Individuals with ASD show considerable variation in the severity and extent to which they exhibit characteristics of the disorder. This heterogeneity has led some researchers to posit that ASD lies on a continuum of social-communication difficulties [17], [18] that extends into the ‘typical’ population [30]. Consistent with this hypothesis, behavioural studies of typical participants have shown effects of the spectrum of autism traits on tasks that are impaired in ASD [19], [31], [32].

The relationship between the STS and ASD has been examined repeatedly in structural MRI and functional neuroimaging studies performed in the resting state using single-photon emission computed tomography (SPECT) and positron emission tomography (PET). In an MRI study using voxel-based morphometry, Levitt et al. [33] investigated anatomic shifting of the STS in an autistic group compared with a normal group, and Boddaert et al. [34] observed that autism is associated with bilateral anatomical abnormalities localized in the STS. Using SPECT in the resting state, Ohnishi et al. [35] reported decreases in rCBF in the superior temporal and prefrontal cortices in autistic patients compared with controls. Furthermore, von dem Hagen et al. [30] recently identified a relationship between autistic traits in the typical (non-ASD) population and the STS using MRI.

Moreover, fMRI activation studies using several social cognitive stimuli have demonstrated impaired STS function in high-functioning ASD subjects under several conditions: bilaterally in a face perception task [36], a voice perception task [37], and a theory-of-mind task [4], as well as right STS dysfunction in a face perception task [2] and an eye-gaze processing task [38]. Our finding of hypoactivation in the STS in subjects with higher autistic trait scores is consistent with these previous findings.

However, in our study, autistic trait scores correlated with brain activation only in the left STS. We consider that this result may be due to the task characteristics in this study. Because the conversation consisted of speaking, i.e. a language function, effects only in the left STS could be due to hemisphere laterality, although the probes were not located in language regions. However, 2 previous PET studies have also shown left STS dysfunction in ASD. Meresse et al. [39] found a significant negative correlation between rCBF in the left superior temporal gyrus and the Autism Diagnostic Interview-Revised scores of autistic patients in the resting state, suggesting that left superior temporal area hypoperfusion is related to the severity of autistic traits. Boddaert et al. [34] observed left temporal area hypoperfusion in autistic patients by PET during a complex auditory processing task.

### Gender and Autistic Traits

In this study, a significant correlation between brain activation and autistic trait scores was only observed in the left STS in male subjects. Gender differences in such a correlation appear reasonable because the prevalence of autism is overwhelmingly higher in males than in females, and the prevalence of autistic traits in healthy populations is higher in males than in females [40], although there was no gender difference in AQ in our subjects. In addition, Baron-Cohen [25] has proposed the ‘extreme male brain’ theory of autism, which hypothesizes that autism can be considered an extreme of the normal male profile. The gender differences in the correlations between brain activation and autistic traits observed in the present study may support this theory. However, it is important to note that during the face-to-face conversations, the interviewers in this study were all male. The male gender of the interviewer may have had differential effects on brain activation in male and female participants, because, for example, brain activation has been demonstrated to vary according to the gender of the face presented in an fMRI study using a facial perception task [41].

### Limitations and Future Directions

There were some methodological limitations in this study. First, the sample size was small, and the participants were all medical students or medical interns. This raises the possibility that the participants were not representative of the general population, because AQ scores differ depending on affiliation, e.g. science students score significantly higher than do humanities students [19]. Most medical students in Japan take science courses; therefore, there may have been sampling effects in this study.

Second, although the task design had some limitations as an exact social cognitive task and was unsatisfactory for reproducing an ordinary social-communication situation, it was sufficient for a preliminary investigation of social cognition in a realistic task setting. To eliminate qualitative and quantitative differences in conversation between participants, we imposed unnatural regulations (such as a conversation cycle of 15 s) and a limited topic of conversation (food). Future studies would benefit from a more sophisticated task design or alternative experimental conditions that limit some social cognitive modules, such as eye-gaze or face perception of the subjects, or that specify features such as specific emotional loading or situational context.

Third, NIRS has some methodological limitations, including low spatial resolution (about 3 cm), and it lacks the ability to assess deep brain structures. We chose the STS as the region of interest corresponding to the 5 Chs shown in Figure S1 (online). This region also includes areas around the lateral upper temporal region, i.e. the superior temporal gyrus, temporal pole, and STS. Thus, there remains the possibility of detecting activation of other parts of the lateral upper temporal region in addition to the STS. Strictly speaking, the pathological substrate of ASD and the brain area responsible for social cognition is considered to be the medial PFC, not the entire PFC. However, it is impossible to distinguish the medial PFC from the other areas of the PFC because of the low spatial resolution of the NIRS technique. Thus, it is reasonable to argue that in the present study, the main functions of the PFC were executive functions, which correspond to the dorsolateral PFC. In the future, when NIRS achieves finer spatial resolution, it will be desirable to distinguish between activation of the medial PFC and the dorsolateral PFC. In addition, the probes in this study covered only the region around the frontal area. Involvement of other cortical areas and deep brain structures could not be determined.

## Supporting Information

Figure S1 **Near-infrared spectroscopy (NIRS) positioning and diagram of light sources, light detectors, and channels.** **Upper:** The NIRS probe on the head (right) and sensor allocations on a probe (left). Red indicates a near-infrared light source, white indicates a near-infrared light detector, and green indicates an NIRS measurement channel. **Lower:** The locations of the NIRS channels were probabilistically estimated and anatomically labelled in the standard brain space according to Tsuzuki et al. [27]. We identified 4 regions of interest: the right prefrontal cortex (PFC) for the 5 channels located in the right prefrontal lobe, the right superior temporal sulcus (STS) for the 5 channels located in the right temporal lobe, the left PFC for the 5 channels located in the left prefrontal lobe, and the left STS for the 5 channels located in the left temporal lobe.
(TIF)

Table S1 **Characteristics of Participants.** Characteristics of participants and behavioural data.
(XLSX)
