# Supplementary figures and images for: Autistic Traits and Brain Activation during Face-to-Face Conversations in Typically Developed Adults
Source: PLoS One. 2011 May 27;6(5):e20021. doi: 10.1371/journal.pone.0020021 (PMC3103507; doi:10.1371/journal.pone.0020021)

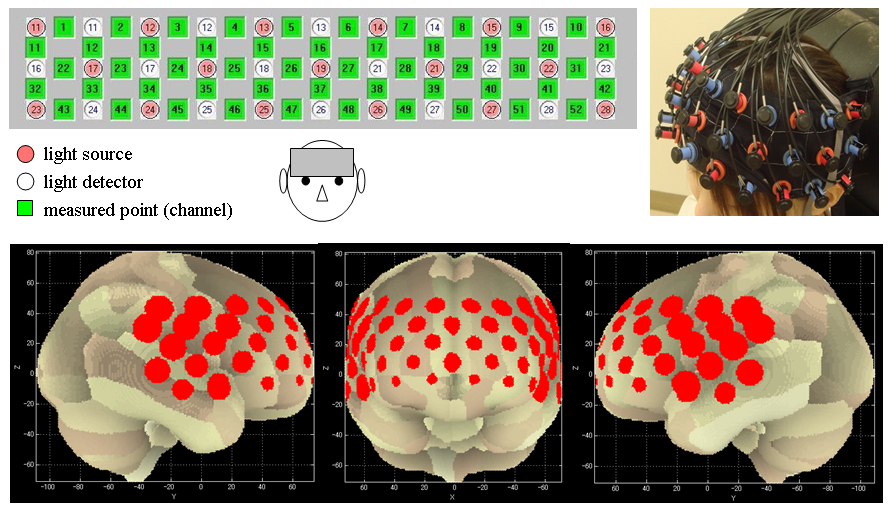

Supplement: Figure S1 — Near-infrared spectroscopy (NIRS) positioning and diagram of light sources, light detectors, and channels. Upper: The NIRS probe on the head (right) and sensor allocations on a probe (left). Red indicates a near-infrared light source, white indicates a near-infrared light detector, and green indicates an NIRS measurement channel. Lower: The locations of the NIRS channels were probabilistically estimated and anatomically labelled in the standard brain space according to Tsuzuki et al. [27]. We identified 4 regions of interest: the right prefrontal cortex (PFC) for the 5 channels located in the right prefrontal lobe, the right superior temporal sulcus (STS) for the 5 channels located in the right temporal lobe, the left PFC for the 5 channels located in the left prefrontal lobe, and the left STS for the 5 channels located in the left temporal lobe. (TIF) [file pone.0020021.s001.tif]
